# Is macular lymphocytic arteritis limited to the skin? Long-term follow-up of seven patients^☆^^☆☆^

**DOI:** 10.1016/j.abd.2019.05.001

**Published:** 2019-11-22

**Authors:** Thâmara Cristiane Alves Batista Morita, Gabriela Franco Sturzeneker Trés, Paulo Ricardo Criado

**Affiliations:** a Department of Dermatology, Hospital das Clinicas, Faculdade de Medicina, Universidade de São Paulo, São Paulo, SP, Brazil; b Faculdade de Medicina do ABC, Santo André, SP, Brazil

**Keywords:** Lipoprotein(a), Livedo reticularis, Polyarteritis nodosa, Skin, Thrombosis, Vasculitis

## Abstract

**Background:**

Macular lymphocytic arteritis most commonly presents as hyperpigmented macules on the lower limbs. The pathogenesis of this disease is still unclear and there is an ongoing debate regarding whether it represents a new form of cutaneous vasculitis or an indolent form of cutaneous polyarteritis nodosa.

**Objective:**

To describe clinical, histopathological, and laboratory findings of patients with the diagnosis of macular lymphocytic arteritis.

**Methods:**

A retrospective search was conducted by reviewing cases followed at the Vasculitis Clinic of the Dermatology Department, School of Medicine, University of São Paulo, between 2005 and 2017. Seven patients were included.

**Results:**

All cases were female, aged 9–46 years, and had hyperpigmented macules mainly on the legs. Three patients reported symptoms. Skin biopsies evidencing a predominantly lymphocytic infiltrate affecting arterioles at the dermal subcutaneous junction were found, as well as a typical luminal fibrin ring. None of the patients developed necrotic ulcers, neurological damage, or systemic manifestations. The follow-up ranged from 18 to 151 months, with a mean duration of 79 months.

**Study limitations:**

This study is subject to a number of limitations: small sample of patients, besides having a retrospective and uncontrolled study design.

**Conclusions:**

To the best of the authors’ knowledge, this series presents the longest duration of follow-up reported to date. During this period, none of the patients showed resolution of the lesions despite treatment, nor did any progress to systemic vasculitis. Similarities between clinical and skin biopsy findings support the hypothesis that macular lymphocytic arteritis is a benign, incomplete, and less aggressive form of cutaneous polyarteritis nodosa.

## Introduction

The first description of macular lymphocytic arteritis (MLA) was made by Fein et al. in 2003.^1^ It is characterized by erythematous or hyperpigmented macules, which can be round, annular, linear, or most often reticulated, affecting mainly the lower limbs, and to a lesser extent the upper extremities and the trunk.^2^, ^3^, ^4^ Nodules are not a typical feature; however, in some cases subtle subcutaneous indurations have been observed.^3^, ^5^, ^6^ Livedo racemosa may appear as an isolated clinical manifestation or along with other dermatologic findings.^5^, ^7^ The lesions follow an indolent course and do not evolve toward a systemic disease, although there have been reports of cutaneous ulceration, testicular infarcts, and neuropathy.^8^, ^9^, ^10^, ^11^

Histologically, MLA shows a dense infiltrate of mononuclear cells in the muscular wall of arterioles at the dermo-subcutaneous junction, with variable narrowing of their lumen by a typical hyalinized fibrin ring.^12^, ^13^ Some authors prefer to refer to it as “lymphocytic thrombophilic arteritis” to highlight the presumed pathogenic mechanism behind this entity.^7^ In fact, it is questionable whether MLA has sufficient defining features to be considered a distinct vasculitis from cutaneous polyarteritis nodosa (C-PAN), a necrotizing arteritis of medium-sized vessels with a predominant polymorphonuclear cell infiltrate.^14^

The aim of the present study was to retrospectively review a series of patients given a diagnosis of MLA to analyze the associated clinical, histopathological, and laboratorial features.

## Methods

The study was approved by the Faculdade de Faculdade de Medicina, Universidade de São Paulo Ethics Committee and all participants gave written informed consent. A total of seven patients diagnosed with MLA between February 2005 and September 2017 at the Vasculitis Clinic of the Dermatology Department were included. Clinical charts were reviewed to obtain additional information, such as sex, race, age at onset of the disease, site of involvement, associated symptoms, and past medical history including thrombotic clinical events. At the time of diagnosis, two skin biopsies of sufficient depth to include subcutis were performed on different occasions for each patient as defined by institutional protocol. For this study, histologic features were reviewed by two experienced dermatopathologists in each case. Laboratory evaluation consisted of complete blood count, renal and liver profile, erythrocyte sedimentation rate, C-reactive protein, urinalysis, complement levels, serum protein electrophoresis, serologic studies for hepatitis B and C, antinuclear antibody (ANA) test, rheumatoid factor (RF), anti–double stranded DNA antibodies, SS-A antibody, SS-B antibody, and antineutrophil cytoplasmic antibodies. A limited thrombophilia screen included the following: lupus anticoagulant; anticardiolipin antibodies (aCL) (immunoglobulin [Ig] M and IgG); anti-β2 glycoprotein I antibodies; tests for protein C and protein S function; factor V Leiden mutation; prothrombin G20210A mutation; and serum levels of antithrombin III, factor VIII, factor IX, fibrinogen, homocysteine, and lipoprotein(a) (Lp[a]). Additional propaedeutic studies with duplex ultrasound of the abdominal aorta, iliac, and renal arteries were carried out in three patients (Patients 4, 5, and 6), as well as duplex ultrasound of the venous system in two patients (Patients 5 and 6) and electromyography in one patient (Patient 6).

For the literature review, in September 2018 the PubMed, Web of Science, and Scopus databases were searched using the terms “macular lymphocytic arteritis,” “macular arteritis,” or “lymphocytic thrombophilic arteritis.” Additional relevant articles were identified by manual inspection of reference lists. Two investigators independently screened the titles and abstracts to determine the potential usefulness of the articles. In all, 34 papers were analyzed. After excluding one study lacking complete data, we summarized the results of 23 articles identified as case reports or case series. They will be further discussed in this article.

## Results

**Figure 1:**
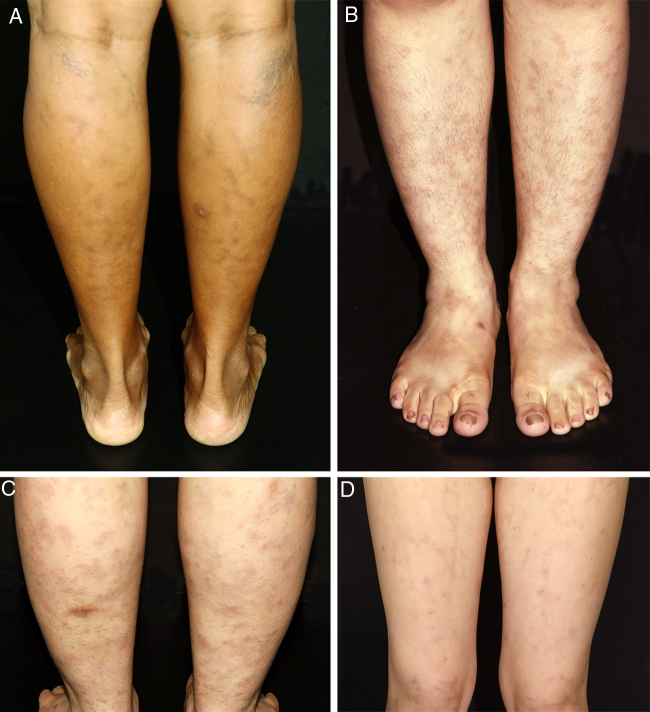
Clinical presentation of macular lymphocytic arteritis over the lower extremities. (A) Multiple linear and sometimes whirled hyperchromic macules, varicose veins, and a biopsy scar in the right calf; (B) rounded and ill-defined macules scattered over the limbs; (C) ill-defined hyperchromic macules, with some lesions being slightly infiltrated on palpation, and a biopsy scar in the left calf; (D) erythematous macules in a reticulated pattern associated with livedo racemosa.

All patients in this series were women. They were classified according to race/skin color. Six patients were white and one was black. Age ranged from 9 to 46 years, with a mean age at the time of diagnosis of 26.71 years (SD = 12.44 years). The duration of the disease before it was diagnosed ranged from 6 to 96 months, with a mean duration of 34.42 months (SD = 32.88 months). Persistent reticular hyperpigmentation was found in all patients; followed by livedo racemosa (two patients), and an equal frequency of erythematous patches and subtle subcutaneous indurations (one patient each; Fig. 1). There was no evidence of ulceration, atrophie blanche, nodules, or palpable purpura. The legs were affected in all patients, while the trunk and arms were affected in one patient each. Lesions presented symmetrically when they affected the limbs.

Symptoms occurring in the same body segment as cutaneous lesions were found in three patients. One of them (Patient 6) complained about paresthesia and pain; for this reason an electromyography was performed and resulted normal, while an ultrasound study of the lower extremities venous system revealed chronic venous insufficiency, due to primary reflux of the great saphenous vein in the left limb. Duplex ultrasound of abdominal aorta, iliac, and renal arteries of Patients 4, 5, and 6 resulted normal, as did duplex ultrasound of the lower extremities of the venous system of Patient 5. None had past history of arterial or venous thrombosis. One of the patients was a former smoker, diagnosed and treated for pulmonary tuberculosis four years before admission. Specific evaluation with examination of sputum by direct smear and chest computed tomography scan showed no signs of active disease. Two patients were given a diagnosis of migraine and followed up by the hospital's neurology team. Demographic data, histopathological findings, treatment, and duration of follow-up of the patients are summarized in table 1.

**Table 1:** Epidemiological features and clinical characteristics of the seven patients with macular lymphocytic arteritis.

Case no.	Sex/age at onset (yrs)	Race/skin color	Previous medical history	Morphology	Duration of disease before diagnosis (months)	Location	Local symptoms	Treatment	Follow-up (months)
1	F/23	White	Migraine	Hyperpigmented macules	96	LL	Arthralgia	ASA HCQ	92
2	F/25	White	–	Hyperpigmented macules, erythematous patches	60	LL + T	None	ASA HCQ	62
3	F/9	White	Migraine	Hyperpigmented macules, livedo racemosa	18	LL	None	ASA	149
4	F/29	White	–	Hyperpigmented macules	6	LL + UL	None	ASA PTX HCQ	41
5	F/17	White	–	Hyperpigmented macules, subtle subcutaneous indurations	12	LL	Pain	ASA PTX HCQ	16
6	F/46	Black	Ex-smoker Pulmonary tuberculosis	Hyperpigmented macules	13	LL	Pain Paresthesia	ASA PTX	41
7	F/38	White	–	Hyperpigmented macules, livedo racemosa	36	LL	None	ASA PTX	146

F, female; LL, lower limbs; T, trunk; UL, upper limbs; ASA, acetylsalicylic acid; HCQ, hydroxychloroquine; PTX, pentoxifylline.

**Figure 2:**
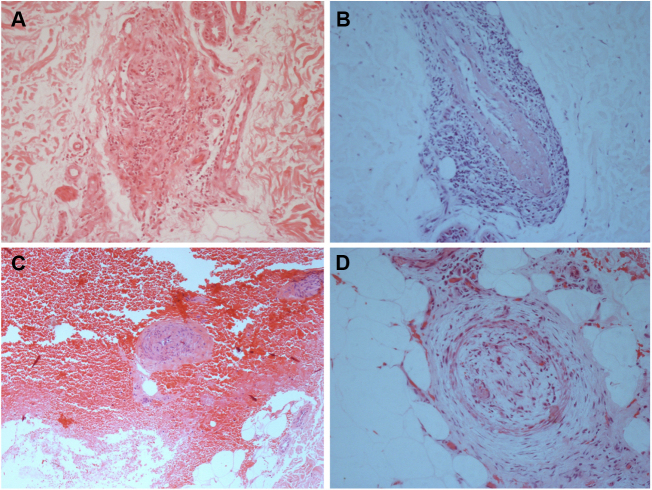
Histological biopsy examination of lesions compatible with lymphocytic macular arteritis in the legs. (A) A small cutaneous artery in the deep dermis is surrounded and infiltrated by lymphocytes (Hematoxylin & eosin, x200); (B) narrowing of the vessel lumen by subintimal thickening (Hematoxylin & eosin, x100); (C) dense inflammation is present around and involving the walls of an arteriole in the superficial subcutaneous layer (Hematoxylin & eosin, x200); (D) hypodermal vessel with organized thrombus (Hematoxylin & eosin, x200).

The laboratory work-up showed few abnormalities, such as a low protein S activity of 28% (reference values: 55–160%; Patient 3); a low level of ANA (1:80) with a nuclear fine speckled pattern and a slightly elevated level of fibrinogen (406 mg/dL; reference values: 150–400 mg/dL; Patient 5). Two patients had positive titers of aCL IgM antibodies at repeated tests (Patients 1 and 3), one of whom also had a positive titer of RF, at 41.7 IU/mL (reference value: <20 IU/mL; Patient 1); and Lp(a) was elevated in three patients, at 107, 75, and 50 mg/dL (reference value: <30 mg/dL; Patients 2, 4, and 6). Histological examination of skin biopsies showed similar findings in all specimens: unremarkable epidermis; lymphocytic infiltrate around small arteries at the deep reticular dermis and superficial fat, and a hyalinized fibrin ring in the vessel lumen in all seven cases; additionally, the vascular lumens were occluded by fibrin thrombi in two patients (Fig. 2). The muscular composition of the medium-sized vessel was best seen with resorcin-fuchsin stain, which revealed a focally discontinuous and disrupted internal elastic lamina in one patient. Neutrophils were not present. Direct immunofluorescence was performed in three patients, and in only one, it revealed C3 deposits in the walls of the dermal blood vessels. Immunohistochemistry study with anti-BCG antibodies was conducted in two patients and resulted normal.

Drug treatment was used alone or in combination, and included acetylsalicylic acid in all seven patients, besides pentoxifylline and hydroxychloroquine in four patients each. Compression stockings and phlebotonics (diosmin and hesperidin) were prescribed to one patient to ameliorate symptoms related to chronic venous insufficiency. Reticular hyperpigmentation expanded to the upper limbs during the use of acetylsalicylic acid and pentoxifylline in one patient. The follow-up ranged from 18 to 151 months, with a mean duration of 79 months. During this period, none of the patients showed resolution of the lesions, nor did any progress to systemic vasculitis.

## Discussion

These results are in accordance with the available literature reviewed. The main clinical presentation of MLA was chronic and persistent hyperpigmented macules, although others dermatologic findings such as livedo racemosa, erythematous patches, and subtle subcutaneous indurations were detected. There was a predilection for the lower extremities. Nevertheless, the frequency of reported symptoms in this series was higher than in previous reports. The clinical characteristics of previously reported patients as well as significant laboratory findings in each case are summarized in the supplementary material (Appendix A).

There were isolated case reports associating MLA to concurrent HIV and hepatitis B infection, rheumatoid arthritis, discoid lupus erythematosus, and to medications or drugs such as minocycline and tobacco.^6^, ^12^, ^15^, ^16^, ^17^, ^18^ Various abnormal laboratory findings have been described, but the present study is the first to report elevated levels of Lp(a) in three patients diagnosed with MLA. Lp(a) is involved in the modulation of platelet aggregation, impairment in fibrinolysis, recruitment of inflammatory cells, and induction of vascular remodeling.^19^ Similarly, the present study found Lp(a) to be elevated in patients with livedoid vasculopathy, a thrombo-occlusive vasculopathy of dermal vessels that usually presents recurrent reticulated purpura on the legs and feet, which lead to ulcerative lesions and atrophic, porcelain-white stellate scars, known as atrophie blanche.^20^

Two patients in the current series had history of migraine with aura. In similar cases, it is essential to exclude Sneddon's syndrome, a non-inflammatory thrombotic vasculopathy clinically characterized by the association of livedo appearance, which involves the trunk and/or buttocks, and ischemic cerebrovascular events, since half of these patients have migraine headache.^21^ Both had two skin biopsies on different occasions each and clinical follow-up longer than five years. One also presented positive titers of aCL IgM antibodies in repeated tests performed at least 12 weeks apart. Though she had no history of thrombocytopenia, past thrombotic event, or obstetric manifestations, it was deemed necessary to rule out antiphospholipid syndrome. Classically, histologic findings in this syndrome are those of thrombosis without significant evidence of inflammation in the vessel wall.^22^

**Figure 3:**
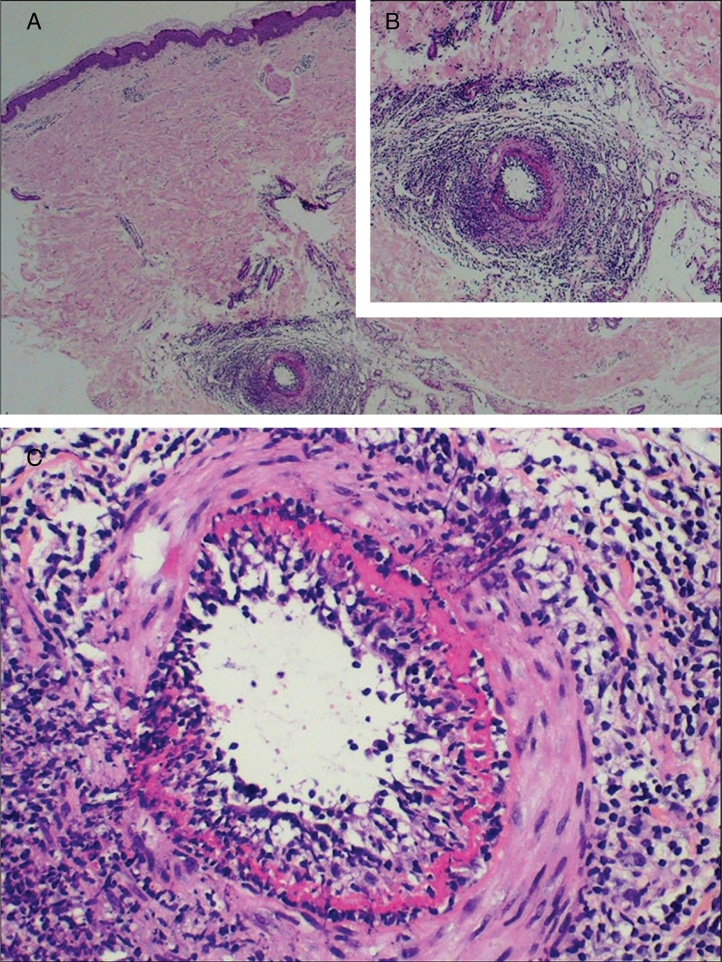
Macular lymphocytic arteritis typical findings. (A) A dermohypodermal junction artery is surrounded by a dense lymphocytic infiltrate that permeates its wall; (B) higher magnification of the previous vessel; (C) a concentric hyalinized fibrin ring is shown (Hematoxylin and eosin, x400).

Typical histological findings were seen in all skin biopsies performed in this group (Fig. 3), including direct immunofluorescence studies negative for vascular or basement deposits of IgG, IgM, and IgA. As previously reported and contrary to the original description, in some cases elastic tissue stain reveals internal elastic lamina disruption.^15^, ^23^, ^24^ One of the histological differential diagnoses of MLA is Buerger disease (thromboangiitis obliterans), a non-atherosclerotic segmental inflammatory pathology that frequently affects small and medium-sized arteries, veins, and nerves in the upper and lower extremities. Essential to the diagnosis is the history of smoking, current or past, as the main etiological agent of the disease.^25^, ^26^ Although one of the patients here described had a history of tobacco use, the absence of changes in skin coloration and temperature of the affected limbs, ischemic ulceration, and gangrene disfavor this diagnosis.

Acute C-PAN lesions, the cutaneous limited form of polyarteritis nodosa, exhibit fibrinoid necrotizing vasculitis of the small and medium-sized arteries at the dermal–subcutis junction, with an infiltrate mainly composed of polymorphonuclear cells. Clinically, C-PAN most often presents as painful subcutaneous nodules and ulcers in the lower legs, usually in association with livedo racemosa. Extracutaneous symptoms, such as peripheral neuropathy, myalgia, and arthralgia, are limited to the same area as skin lesions.^27^, ^28^, ^29^ Relevant characteristics of both MLA and C-PAN are presented in table 2.

**Table 2:** Clinical and histological features of macular lymphocytic arteritis and cutaneous polyarteritis nodosa.

	Macular lymphocytic arteritis	Cutaneous polyarteritis nodosa
Clinical features	Round, linear, or reticular hyperpigmented macules and patches	Palpable purpura, painful subcutaneous nodules, recurrent ulcers, and rarely gangrene and digital necrosis
	Livedo racemosa	Livedo racemosa
	Lesions are asymptomatic to minimally pruritic or painful. Systemic symptoms have been rarely described (testicular infarcts and neuropathy)	Extracutaneous involvement may manifest as myalgia, peripheral neuropathy, and arthralgia, limited to the same area as skin lesions, sometimes accompanied by fever and weight loss
	LL > UL > trunk	LL > UL > trunk
Histological features	Lymphocytic infiltration in the muscular wall of small arteries. Neutrophils are absent or scarce	Leukocytoclastic vasculitis affecting the walls of medium-sized arteries and arterioles of septae in the upper portions of the subcutaneous fat
	Associated fibrinoid necrosis	Associated fibrinoid necrosis in active lesions
	Variable narrowing of the vascular lumen by concentric fibrin deposition and intimal proliferation	Later in the disease process the infiltrate is predominantly composed of lymphocytes and histiocytes, and neoangiogenesis becomes apparent
	Absence of elastic lamina disruption in most cases	Fragmented and discontinuous internal elastic lamina
	Direct immunofluorescence studies are negative for vascular or basement deposits of IgG, IgM, IgA, and C3	Direct immunofluorescence can provide positive results for IgM and C3, either alone or combined
Prognosis	Chronic and benign course	Chronic course, progression from C-PAN to idiopathic generalized PAN rarely occurs

C-PAN, cutaneous polyarteritis nodosa; LL, lower limbs; PAN, systemic polyarteritis nodosa; UL, upper limbs.

Several treatments for MLA have been reported, with variable results. Topical steroids have been tried without any clinical improvement,^15^, ^24^, ^30^ Moreover, patients have not responded to low-dose aspirin, clopidogrel, and warfarin. In fact, in two cases the lesions progressed despite their use. Improvement in the appearance of skin lesions of up to 80% was demonstrated with dapsone 100 mg/day and total resolution after using prednisone in low doses, methotrexate, and colchicine, though the follow-up duration reported in this case was only eight months.^30^, ^31^, ^32^ The present authors chose acetylsalicylic acid and pentoxifylline as first-line treatments for MLA. In four of the current patients, hydroxychloroquine was added due to persistence of symptoms or extension of the lesions beyond the legs. The patients remained stable. No regression of the cutaneous macular lesions was observed, nor appearance of necrotic ulcers, neurological damage, or systemic manifestations. To the best of the authors’ knowledge, the mean follow-up duration of this series of patients is the longest to date.

## Conclusions

In short, this study described seven patients whose clinical and histopathological presentation is compatible with MLA. Given the overlap in clinical features, there is still significant debate regarding whether or not MLA could be a precursor or an indolent form of C-PAN. In fact, considering that there is a well-known transition from a predominant neutrophilic to a lymphocytic infiltrate in the subacute and reparative stages in C-PAN, and that some patients with MLA may exhibit intermediate histologic features, such as fibrinoid necrosis and internal elastic lamina disruption, it may be deemed reasonable to consider that these two entities belong to the same disease spectrum. The authors propose that MLA may represent a less aggressive immune response in the arterial component of mid-diameter cutaneous vessels, which is insufficient to provoke ulcerations, scarring, or very troublesome symptoms in most of these cases. Over time, further research on etiopathogenesis may clarify the relationship between them.

## Financial support

None declared.

## Authors’ contribution

Thamara Cristiane Alves Batista Morita: Statistical analysis; approval of the final version of the manuscript, composition of the manuscript; collection, analysis, and interpretation of data; intellectual participation in the propaedeutic and/or therapeutic conduct of the studied cases; critical review of the literature; critical review of the manuscript.

Gabriela Franco Sturzeneker Trés: Approval of the final version of the manuscript; composition of the manuscript; intellectual participation in the propaedeutic and/or therapeutic conduct of the studied cases; critical review of the literature.

Paulo Ricardo Criado: Approval of the final version of the manuscript; conception and planning of the study; participation in the study design; intellectual participation in the propaedeutic and/or therapeutic conduct of the studied cases; critical review of the manuscript.

## Conflicts of interest

None declared.
