# Supplementary material for: Is macular lymphocytic arteritis limited to the skin? Long-term follow-up of seven patients
Source: An Bras Dermatol. 2019 Nov 22;95(1):32–9. doi: 10.1016/j.abd.2019.05.001 (PMC8074687; doi:10.1016/j.abd.2019.05.001)
Supplement: Supplementary file 1 [file mmc1.docx]

**Supplementary material**

Reported cases of macular lymphocytic arteritis in the literature.

| **Reference** | **Case** | **Age (yrs)** | **Gender** | **Race/Origin** | **Duration of disease before diagnosis** | **Hyperpigmented macules** | **Others clinical features** | **Distribution** | **Symptoms and other complaints** | **Associated conditions** | **Follow-up** |
| --- | --- | --- | --- | --- | --- | --- | --- | --- | --- | --- | --- |
| Fein H, Sheth AP, Mutasim DF, 2003^1^ | 1 | 17 | F | African-American | 5 years | Present | Patches | UL + LL + T | Mild pruritus | None | Not reported |
|  | 2 | 73 | F | African-American | 6 months | Present | None | UL + LL | Mild pruritus | Hypertension, asthma, glaucoma | 18 months |
|  | 3 | 27 | M | African-American | 15 years | Present | Patches | UL + LL | None | None | Not reported |
| Sadahira C, et al., 2005^2^ | 4 | 49 | M | Japanese | 4 years | Present | None | UL + LL | None | Heavy smoker | 5 years |
|  | 5 | 52 | F | Japanese | 4 months | Present | None | UL + LL | None | Heavy smoker | Not reported |
| Al-Daraji W, et al, 2008^3^ | 6 | 47 | M | Not reported | 2 months | Present | Scaly papules | Left LL | None | Asthma and osteoarthritis | 6 months |
| Lee JS, et al, 2008^4^ | 7 | 27 | F | Chinese | 2 years^a^ | Absent | SIL, nodules, livedo | UL + LL | Pain | None reported | ≥ 7 months |
|  | 8 | 23 | F | Middle Eastern | 2 years | Absent | SIL, livedo | UL + LL | Peripheral numbness | Smoker, Raynaud phenomenon | ≥ 8 months |
|  | 9 | 21 | F | Iraqi | 3 years | Absent | SIL, livedo | UL + LL | None | Headache, mutation of the factor V Leiden gene | ≥ 6 months |
|  | 10 | 20 | F | Mixed Japanese and English | 4 years | Absent | SIL, livedo | UL + LL | None | None | 10 months |
|  | 11 | 34 | F | Indian | 1 year | Absent | SIL, livedo | LL | Muscle weakness | None | Defaulted follow-up |
| Buckthal-McCuin J, Mutasim D, 2009^5^ | 12 | 6 | F | Caucasian | 4 months | Absent | Hyperpigmented patches, petechiae | Left LL | Mild pruritus | Asthma, bipolar disorder | Not reported |
| Saleh Z, Mutasim DF, 2009^6^ | 13 | 30 | F | Mixed Asian and African-american | 2 years | Present | None | LL | None | Hypothyroidism | Not reported |
|  | 14 | 59 | F | African-American | 7 months | Absent | Hypopigmented patches | LL | None | DLE, hypertension, CAD, and alcoholic fatty liver | Not reported |
|  | 15 | 50 | F | African-American | 1 week | Absent | Hyperpigmented patches | LL | None | DLE, cardiac arrhythmias, and depression | Not reported |
| Morruzzi C, et al., 2010^7^ | 16 | 39 | F | Tunisian | 3 years | Present | None | LL | None | None | 12 months |
| Gupta S, et al, 2011^8^ | 17 | 28 | F | Costa Rican | 6 years | Absent | Livedo | UL + LL | None | None | 9 months |
| Munehiro A, et al, 2012^9^ | 18 | 56 | M | Japanese | Not reported | Present | None | LL | None | Rheumatoid arthritis | Not reported |
| Kassardjian M, et al, 2012^10^ | 21 | 40 | F | Caucasian | 3 months | Absent | Hyperpigmented patches | UL + LL | None | None | Not reported |
| Valverde R, et al, 2013^11^ | 20 | 61 | F | Not reported | 3 years | Present | None | LL | None | None | 1 year |
| Macarenco RS, et al, 2013^12^ | 19 | 19 | F | Not reported | 4 years | Present | Papules, livedo, stellate white scars | LL | Mild pain | None | 1 year |
| Llamas-Velasco M, et al, 2013^13^ | 22 | 25 | F | Hispanic | 1 year^b^ | Absent | Patches, petechiae, ulcers | LL | None | None | 42 months |
| Shen S, et al, 2013^14^ | 23 | 35 | F | Lebanese | 3 years | Absent | Livedo | UL + LL | LL numbness and weakness | Heterozygous for prothrombin gene mutation | Not reported |
| Garcia C, et al, 2014^15^ | 24 | 43 | F | North African | 1 year | Present | None | LL | None | Hashimoto’s thyroiditis, hypertension | 3 years |
|  | 25 | 38 | F | North African | 1 year | Present | SIL | LL | None | Raynaud phenomenon | 2 years |
|  | 26 | 53 | F | North African | 10 months | Present | None | LL | None | Oral canker sores, hypertension | 6 months |
| Kalegowda IY, et al, 2014^16^ | 27 | 53 | F | Indian | 5 years | Present | Patches, SIL, Livedo | UL + LL | Mild pruritus and pain | None | 6 months |
| Kolivras A, et al, 2015^17^ | 28 | 33 | F | African | 10 years | Present | SIL | UL + LL + T | None | HBV, HIV | Not reported |
| Taconet, S, et al, 2015^18^ | 29 | 59 | M | Not reported | 1 year | Present | None | UL + LL | None^c^ | Chronic venous insufficiency | Not reported |
| Védie AL, et al, 2016^19^ | 30 | 62 | F | Not reported | 5 years | Present | Livedo | LL | None | None | 5 years |
|  | 31 | 61 | M | Not reported | 4 months | Present | Livedo | LL | None | None | 4 months |
|  | 32 | 66 | F | Not reported | 1 year | Present | None | LL | None | None | 1 year |
| Arana-Guajardo A, et al, 2016^20^ | 33 | 23 | F | Not reported | 8 weeks | Present | None | Right UL + LL | None | None | 8 months |
| Zampella JG, et al, 2017^21^ | 34 | 34 | F | African-American | 1 year | Absent | Patches | LL | None | NR | 9 months |
|  | 35 | 60 | F | White | Not reported | Present | Papules | LL | None | None | 6 months |
|  | 36 | 44 | F | Nigerian | Not reported | Present | Patches | LL | Mild pruritus | DM | 4 months |
|  | 37 | 60 | F | White | Not reported | Present | Patches | LL | None | Coronary vasospasm | 2 years |
| Awan SZ, et al, 2017^22^ | 38 | Early 20’s ^d^ | F | Not reported | 1 year | Present | None | LL | None | None | Defaulted follow-up |
| We, E, et al, 2018^23^ | 39 | 26 | M | Caucasian | 1 year | Absent | Livedo | UL + LL + T | None^e^ | Chiari malformation, heterozygosity for prothrombin gene mutation and an only slightly raised rheumatoid factor (28; normal < 14 IU/mL) | >5 years |

^a^The patient had similar lesions 11 years earlier that lasted 2 months; ^b^The patient had similar lesions six years earlier of unspecified duration; ^c^The patient developed paraesthesia of the left upper limb six months after presentation; ^d^Unspecified; ^e^Later, the patient ﻿developed bilateral focal testicular infarcts and an acute median nerve neuropathy.

F: Female; M: Male, SIL: Slightly indurated lesions; UL: Upper limbs; LL: Lower limbs; T: Trunk; DLE: Discoid lupus erythematosus; CAD: Coronary artery disease; HBV: Hepatitis B infection; HIV: Human immunodeficiency virus infection; DM: Diabetes Mellitus

REFERENCES

1. Fein H, Sheth AP, Mutasim DF. Cutaneous arteritis presenting with hyperpigmented macules: Macular arteritis. J Am Acad Dermatol. 2003;49(3):519–22.

2. Sadahira C, Yoshida T, Matsuoka Y, Takai I, Noda M, Kubota Y. Macular arteritis in Japanese patients. J Am Acad Dermatol [Internet]. 2005 Feb;52(2):364–6. Available from: http://linkinghub.elsevier.com/retrieve/pii/S0190962204021760

3. Al-Daraji W, Gregory a N, Carlson JA. “Macular arteritis”: a latent form of cutaneous polyarteritis nodosa? Am J Dermatopathol. 2008;30(2):145–9.

4. Lee JS, Kossard S, McGrath MA. Lymphocytic Thrombophilic Arteritis. Arch Dermatol [Internet]. 2008 Sep 1;144(9):1175–82. Available from: http://archderm.jamanetwork.com/article.aspx?doi=10.1001/archderm.144.9.1175

5. Buckthal-McCuin J, Mutasim DF. Macular Arteritis Mimicking Pigmented Purpuric Dermatosis in a 6-Year-Old Caucasian Girl. Pediatr Dermatol [Internet]. 2009 Jan;26(1):93–5. Available from: http://doi.wiley.com/10.1111/j.1525-1470.2008.00831.x

6. Saleh Z, Mutasim DF. Macular lymphocytic arteritis: A unique benign cutaneous arteritis, mediated by lymphocytes and appearing as macules. J Cutan Pathol. 2009;36(12):1269–74.

7. Morruzzi C, Cribier B, Lipsker D. Artérite maculeuse. Ann Dermatol Venereol [Internet]. 2010;137(6–7):460–3. Available from: http://linkinghub.elsevier.com/retrieve/pii/S0151963810002140

8. Gupta S, Mar A, Dowling JP, Cowen P. Lymphocytic thrombophilic arteritis presenting as localized livedo racemosa. Australas J Dermatol. 2011;52(1):52–5.

9. Munehiro A, Yoneda K, Koura A, Nakai K, Kubota Y. Macular lymphocytic arteritis in a patient with rheumatoid arthritis. Eur J Dermatology. 2012;22(3):427–8.

10. Kassardjian M, Horowitz D, Shitabata PK, Clark LE. Lymphocytic thrombophilic arteritis induced by minocycline. J Clin Aesthet Dermatol. 2012;5(10):38–43.

11. Valverde R, Garrido C, Leis V, Ruiz-Bravo E. Arteritis macular: ¿en el espectro de la poliarteritis nudosa cutánea? Actas Dermosifiliogr [Internet]. 2013 Apr;104(3):263–5. Available from: http://linkinghub.elsevier.com/retrieve/pii/S0001731012003420

12. Macarenco RS, Galan A, Simoni PM, Macarenco AC, Tintle SJ, Rose R, et al. Cutaneous Lymphocytic Thrombophilic (Macular) Arteritis: A Distinct Entity or An Indolent (Reparative) Stage of Cutaneous Polyarteritis Nodosa? Report of 2 Cases of Cutaneous Arteritis and Review of the Literature. Am J Dermatopathol. 2013;35(2):213–9.

13. Llamas-Velasco M, García-Martín P, Sánchez-Pérez J, Sotomayor E, Fraga J, García-Diez A. Macular lymphocytic arteritis: First clinical presentation with ulcers. J Cutan Pathol. 2013;40(4):424–7.

14. Shen S, Williams RA, Kelly RI. Neuropathy in a patient with lymphocytic thrombophilic arteritis. Australas J Dermatol [Internet]. 2013 May;54(2):e28–32. Available from: http://doi.wiley.com/10.1111/j.1440-0960.2011.00827.x

15. Garcia C, Dandurand M, Roger P, Joujoux JM, Meunier L, Stoebner PE. Macular lymphocytic arteritis: Three cases questioning its classification as primary lymphocytic vasculitis. Dermatology. 2014;228(2):103–6.

16. Tirumalae R, Murthy Ks, Rout P, Kalegowda I. Lymphocytic thrombophilic arteritis: An enigma. Indian J Dermatol [Internet]. 2014;59(5):498. Available from: http://www.e-ijd.org/text.asp?2014/59/5/498/139907

17. Kolivras A, Thompson C, Metz T, André J. Macular arteritis associated with concurrent HIV and hepatitis B infections: A case report and evidence for a disease spectrum association with cutaneous polyarteritis nodosa. J Cutan Pathol. 2015;42(6):416–9.

18. Taconet S, Vignon-Pennamen M-D, Fouchard N. Artérite maculeuse lymphocytaire et périartérite noueuse : un cas illustrant les difficultés diagnostiques et nosologiques posées par ces deux entités. Ann Dermatol Venereol [Internet]. 2015;142(10):567–71. Available from: http://linkinghub.elsevier.com/retrieve/pii/S0151963815004834

19. Védie AL, Fauconneau A, Vergier B, Imbert E, De La Valussière G, Demay O, et al. Macular lymphocytic arteritis, a new cutaneous vasculitis. J Eur Acad Dermatology Venereol. 2016;30(3):542–4.

20. Arana-Guajardo A, Mendoza-Rodríguez C, Miranda-Maldonado I. Unilateral Macular Lymphocytic Arteritis. J Rheumatol [Internet]. 2016;43(9):1763–4. Available from: http://www.ncbi.nlm.nih.gov/pubmed/27587013

21. Zampella JG, Vakili S, Doig S, Girardi N, Kwatra SG, Seo P, et al. Macular lymphocytic arteritis: Clinical-pathologic correlation of a rare vasculitis. JAAD Case Reports [Internet]. 2017;3(2):116–20. Available from: http://linkinghub.elsevier.com/retrieve/pii/S2352512617300176

22. Awan SZ, Finch J, Kristjansson A. An Asymptomatic Hyperpigmented Eruption on the Lower Extremities. JAMA Dermatology [Internet]. 2017;1–2. Available from: http://archderm.jamanetwork.com/article.aspx?doi=10.1001/jamadermatol.2017.0112

23. Wee E, Nikpour M, Balta S, Williams RA, Kelly RI. Lymphocytic thrombophilic arteritis complicated by systemic involvement. Australas J Dermatol [Internet]. 2018;(December 2017):2017–9. Available from: http://doi.wiley.com/10.1111/ajd.12798
